# Automatic myocardium segmentation of LGE MRI by deformable models with prior shape data

**DOI:** 10.1186/1532-429X-15-S1-P14

**Published:** 2013-01-30

**Authors:** YingLi Lu, Graham Wright, Perry E Radau

**Affiliations:** 1 Imaging Research, Sunnybrook research institute, Toronto, ON, Canada; 2 Medical Biophysics, University of Toronto, Toronto, ON, Canada

## Background

Previously a myocardial tissue classification algorithm has been developed to locate and quantify infarct in a given myocardial region-of-interest specified on late gadolinium enhancement (LGE) MR images [1]. To complete the automation requires an endocardial and epicardial contour detection algorithm to replace the current practice of manual contouring that is time-consuming and subject to intra- and inter-observer variability. Challenges include: 1) the intensity inhomogeneity of both the healthy and infarct myocardium; 2) the existence of an infarct on a given slice is not known a priori; 3) a sub-endocardial infarct region's boundary can be easily mistaken for the endocardial contour due to the proximity and strength of the edge (gradient); and 4) incorporating prior anatomical information (e.g., cine steady-state free precession (SSFP) MRI) while allowing for possible motion between separate studies.

## Methods

**Figure 1:**
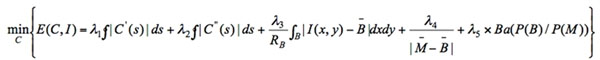
Energy function of the deformable model. C(s)=(x(s),y(s)) is the parameterized contour (endo- and epicardial), P(B) is the histogram of blood pool and P(M) as the histogram of myocardium. Ba(f/g) is the Bhattacharyya coefficient measuring the amount of overlap between two statistical samples f and g. Shape is the Fourier descriptors of the discretized contour. Each contour is constrained by the corresponding cine contour shape, and by myocardial thickness.

The proposed deformable contour algorithm addresses these challenges by minimizing an energy function that: 1) incorporates intensity overlap into the deformable model by applying Bhattacharyya coefficient [2]; 2) eliminates the image gradient term of the energy function; and 3) incorporates hard constraints based upon prior information about contour shape and myocardium thickness from the pre-delineated contours of the corresponding cine SSFP image (see equation in Figure 1). Ten patients with known CAD and chronic MI had cardiac LGE MR scans. Our method uses pre-delineated endo- and epicardial contours from the corresponding cine MRI as a priori knowledge. We registered the corresponding cine MR image to the LGE image using mutual information image registration with affine transformation, then applied the resulting transform to the pre-delineated endo- and epicardial cine MRI contours, to initialize the LGE MRI segmentation. The contours were refined by minimizing the deformable contour energy function locally with greedy optimization [3].

## Results

**Figure 2:**
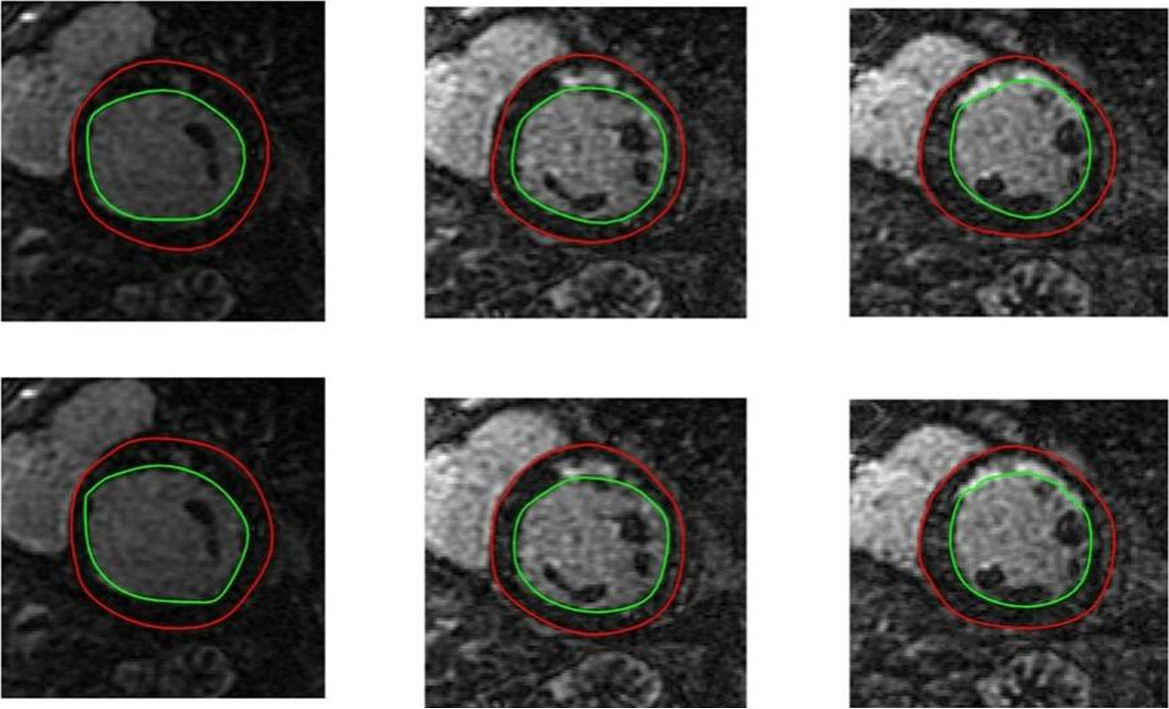
Representative automatic segmentation results (top row) compared to expert results (bottom row).

The average perpendicular distance between manually drawn and automatically detected contours was 1.03 ±0.4 mm and 0.92±0.32 mm for endocardial and epicardial contours, respectively. The average Dice coefficient was 0.95±0.02 for endocardium and 0.97±0.01 for epicardium and 0.85±0.05 for the myocardium. Representative results in Figure 2. By visual examination 19% contours (27/144 contours) required manual corrections. This is a substantial improvement compared with previous rate of about 50% when propagating cine contours without the deformable contour algorithm.

## Conclusions

Our method provides a high degree of automation and accuracy. The results for the proposed automated segmentation technique indicate that it will streamline accurate quantification of myocardial infarct on LGE MR infarct images in clinical practice.

## Funding

MaRS Innovation 2011 Medical Sciences Competitive Proof of Principle (MSCPoP) for Medical Devices and Ontario Research Fund, (Imaging for Cardiovascular Therapeutics)
